# A smartphone application as a personalized treatment tool for adolescents with overweight: an explorative qualitative study

**DOI:** 10.1186/s12889-023-15248-z

**Published:** 2023-04-14

**Authors:** Maurane Desmet, Sieske Franssen, Tugce Varol, Alicia Fillon, David Thivel, Anne Roefs, Caroline Braet

**Affiliations:** 1 Department of Developmental, Personality and Social Psychology, Faculty of Psychology and Educational Sciences, Ghent University, Ghent, Belgium; 2 Department of Clinical Psychological Science, Faculty of Psychology and Neuroscience, Maastricht University, Maastricht, the Netherlands; 3 Department of Work and Social Psychology, Faculty of Psychology and Neuroscience, Maastricht University, Maastricht, the Netherlands; 4 Laboratory of the Metabolic Adaptations to Exercise under Physiological and Pathological Conditions (AME2P), Clermont Auvergne University, Clermont-Ferrand, EA 3533 France; 5 National Observatory for Physical Activity and Sedentary behaviors (ONAPS), Clermont-Ferrand, France

**Keywords:** Overweight, Adolescents, eHealth, App, Personalized, Treatment

## Abstract

**Background:**

The present study is the first step of a 3-year European project in which a tailored smartphone application will be developed and tested as a potential tool in the personalized treatment of children and adolescents with overweight.

**Methods:**

In this study, 10 focus groups (*n* = 48 participants) were conducted in Belgium, The Netherlands and France with adolescents with overweight (12–16 years; *n* = 30) and parents of adolescents with overweight (*n* = 18) to investigate their perceptions on (un)healthy behavior, the drivers of these behaviors, and the needs of an eHealth application for weight loss. A thorough thematic analysis was performed using Nvivo12.

**Results:**

Results show that adolescents with overweight have a well-articulated perspective on (un)healthy behavior and their needs. Parents underestimate their own influence on the (un)healthy behavior of their children and report difficulties in healthy lifestyle parenting, which makes their role as a coach rather ambiguous. Concerning the needs of an eHealth application, both parents and adolescents formulated some challenging expectations regarding the content and the format including information, a monitoring feature and features that increase participants’ motivation to behave healthy. The results of this analysis will form the basis for designing a personalized eHealth application, which will be tested in a next phase.

**Conclusion:**

We can conclude that adolescents have a well-articulated perspective on healthy and unhealthy behavior and their needs, whereby a new app could be of great help. It could function as a day-by-day diary and as a supportive coach.

## Background

The worldwide prevalence of overweight and obesity has risen sharply over the last 35 years, in children, adolescents and adults. Statistics of 2016 show that worldwide more than 340 million children and adolescents aged 5–19 are overweight [1, 2]. This ‘obesity epidemic’ is alarming because overweight is associated with numerous negative consequences. In the short term, pediatric overweight is linked with stigmatization and psychosocial distress, including low self-esteem and symptoms of anxiety and depressive disorder [3]. The prevalence of psychiatric disorders among children and adolescents with overweight is strikingly high, with data suggesting that more than 30% of children and adolescents with overweight meet criteria for at least one psychiatric disorder [4]. Additionally, these children and adolescents can encounter physical problems such as sleep apnea, asthma, cardiovascular disease and type 2 diabetes [3, 5]. In the long term, overweight during childhood and adolescence is a significant risk factor for adulthood overweight, which in its turn entails many harmful physical and psychological health risks [6, 7]. Finally, this chronic disease also has major economic consequences, including both direct (healthcare) and indirect (productivity loss) costs. The mean total lifetime cost reaches up to approximately €150.000 per child or adolescent [8, 9].

Evidence shows that the odds of spontaneous remission of pediatric overweight are low [10, 11], making focusing on both its prevention and its treatment of great importance. Currently multidisciplinary interventions of several weeks that combine a dietary approach, exercise, behavioral and cognitive therapy techniques remain the standard [12–14]. Parental involvement is pursued as much as possible in these interventions, as parents are recognized as important actors in the eating and physical activity behavior of the child [15–17]. Although considerable research has been done in recent years, the effectiveness of the current multidisciplinary interventions remains low, with substantial relapse rates. Cochrane meta-analyses of evidence-based interventions reported an average of one-year reductions in BMI z-score of only − 0.06 units in children and only − 0.13 units among adolescents [18, 19]. Existing treatments for obesity could be improved in two ways. Firstly, by elaborating more lifetime follow-up care as this is crucial to maintain control over this chronic disease [6, 20, 21]. Secondly, by incorporating more personalized support as gaining weight and becoming overweight has multicausal origins, resting on a complex interplay of biological, psychological and social factors [22]. This makes obesity a heterogeneous problem with large inter-individual differences, even in younger age groups. However, depending on the underlying specific factors, we could distinguish subtypes in both adults [23] and younger age groups [24, 25] which has already led to some specified subtype related interventions. Research on the added value of personalized support both in the existing treatment programs and in lifetime care has not yet been taken into account and is highly required [18, 19, 26]. A possible solution to (a) identify the specific individual problems each adolescent with overweight faces, and (b) find ways for sustainable support, is mobile eHealth. eHealth refers to the use of information and communication technologies (ICT) in healthcare [27]. Over the last decade, specifically smartphone applications (apps) have been used as tools in numerous mental health treatments [28]. Various studies have demonstrated the added value of such apps to improve mental health by offering a wide range of possible uses, such as psychoeducation, assessment, symptom monitoring, skills training, and tracking treatment progress [28, 29]. Importantly, apps as treatment tools are easily accessible in daily life, proven to be time and cost-efficient and usable over a long time, which makes them sustainable [30, 31]. In addition, monitoring tools can be beneficial to track and gain insights in individual behaviors, attitudes, or emotions in a systematic way. A way to attain this is by using Ecological Momentary Assessment (EMA), a research methodology that involves the repeated sampling of for example people’s current thoughts, feelings, behaviors and environments. In contrast to other assessment methods in psychology, which rely on global retrospective self-reports, EMA relies on reports in real time and in participants’ natural environments. Therefore, EMA minimizes recall bias, maximizes construct and ecological validity and allows to study microprocesses that influence behavior in real world contexts [32, 33]. A mobile application in which EMA was used for the real-time monitoring of person-environment interactions and treatment support of obesity in adults is Think Slim. This eHealth intervention consists of (a) an app-based EMA that estimates when people are likely to eat and intervenes at such moments, and (b) a CBT-based intervention tool that changes patterns of obesity-related dysfunctional thinking and increases self-esteem [26]. However, whether and how this is useful in the treatment of adolescents with overweight is currently still unknown. The present study is the first step of a three-year European project in which a similar app (both as a monitoring tool and as an intervention tool) will be developed and tested as a potential support for both the long-term after-care and the personalized treatment of overweight adolescents.

The integration of eHealth apps in treatment is not without challenges, as it is difficult to maintain a high adherence, especially with longer intervention durations [34, 35]. In a recent study of Browne and colleagues [36], in which the feasibility of an eHealth intervention tool was tested in overweight children and adolescents, only 37% of participants fully adhered to the eHealth treatment protocol. This result emphasizes the need to learn more about the needs of the target group to enhance the engagement in such interventions. Given the challenges with adherence to eHealth, involving the end-users of the app early on in the design phase could shed new light on app requirements. Therefore, the present study aims to gain insights into the needs of the target group, by having focus groups with both adolescents with overweight and their parents. Focus groups enable the gathering of qualitative data on different issues that are of concern to the participants [37]. This study is set up as an explorative qualitative study and investigates (1) which perceptions adolescents with overweight and their parents have regarding (un)healthy behavior, (2) how participants elaborate and reflect on internal and external drivers that are associated with (un)healthy behavior and (3) whether and how adolescents and parents think an app could be a useful tool to achieve healthy behavior. These insights will form the basis for designing the personalized eHealth app.

## Methods

The COREQ checklist [38] was used to report our research.

### Participants

Participants were recruited in October 2020 in three European countries: Belgium, the Netherlands and France, in three different settings: inpatient center, general population not following treatment and outpatient center. In total, 30 adolescents aged 11–16 years and 18 parents of the adolescent participants participated in the study. Inclusion criteria for the adolescents were (1) age between 12 and 16 years, and (2) being overweight; %BMI score ≥ 120% (adjusted BMI for age and gender [39]). Exclusion criteria in all countries were (1) age < 12 years and ≥ 17 years and (2) %BMI score < 120%. In the Netherlands, participants following treatment were excluded as well, to ensure we could reach a group that is not in treatment. In Belgium, five adolescents with overweight were recruited in an inpatient treatment center as part of another study in which they participated. In France, fifteen adolescents with overweight and eight parents were recruited in an outpatient treatment center as part of another study in which they participated. In Belgium and France, the adolescents and parents were approached face-to-face by a clinician of the treatment center. In the Netherlands, ten adolescents with overweight and ten parents were recruited from the community by a recruitment agency via advertisements on the website of the recruitment agency. No one refused to participate or dropped out.

### Procedure

#### Focus groups

Due to COVID-19, all focus group discussions were conducted online in November 2020, in groups of approximately five participants. In Belgium, one focus group was conducted with five adolescents. In the Netherlands, two focus groups of five adolescents and two focus groups of five parents were conducted, and in France, three focus groups of five adolescents and two focus groups of four parents were conducted. This study was approved by the Ethics Committee of the participating universities and in accordance with the Helsinki Declaration.

#### Interview guides

Written informed assent was obtained from adolescents (≥ 12y) as well as informed consent from their parents. Two semi-structured interview guides (one for adolescents and one for parents) were developed by MD (*child & adolescent psychologist, PhD student*) and SF (*Cognitive neuroscientist, PhD*) to ensure standardization across the three European countries. The development of the interview guides was based on the child obesity literature (Braet, 2010) and was done in collaboration with experienced clinical psychologists who treat childhood obesity and experienced qualitative researchers. The interview guides were validated by experienced researchers in three countries: Belgium, the Netherlands and France. The first version of the adolescent interview script was revised, using more child friendly language. The interview guides were developed in English and translated into Dutch and French, using the back translation method. The translated text was translated back into the source language by independent researchers mastering both English and Dutch. The back translation and the original document were compared by the first author for inconsistencies and a consensus was reached by the first two authors [40, 41]. Both adolescents’ and parents’ interview scripts included open questions in four main topics: Topic 1: perceptions of healthy and unhealthy behavior; Topic 2: internal drivers of healthy and unhealthy behavior; Topic 3: external drivers of a healthy and unhealthy behavior and Topic 4: needs in eHealth applications. Prior to the focus groups, the adolescent interview guide was pilot tested in a group of three adolescents to test the comprehensibility and timing of the focus group discussions.

#### Moderators

The focus groups in Belgium and the Netherlands were led by a moderator (MD; *child & adolescent psychologist, PhD student, female*) and a co-moderator (NV; *design researcher, female*). In France, there was only one moderator (AF, *obesity therapist, PhD*, *female*). Each moderator had experience in conducting interviews or was trained in doctoral schools. A detailed protocol describing all steps for the (co)moderator was followed. The moderators and co-moderator made field notes during the focus groups. No relationship was established with the adolescents prior to the focus groups. In Belgium, a clinician of the treatment center also participated in the study to create a safe environment for the adolescents.

#### Protocol

At the start of the focus groups, the study purpose was first explained to the participants and rules were communicated. To increase participant comfort, an ice-breaker activity was provided at the start of the focus groups by the (co)moderator, and each participant introduced themselves. According to the protocol, the (co)moderator did not share personal characteristics that could influence the research. After the introduction, the participants were asked to respond to open questions that were grouped in topics and handled one by one. No alternatives were used when discussing each topic; the adolescents could share their experiences voluntarily. After discussing every topic, participants were asked if they wanted to add something to make sure every adolescent had the opportunity to share their experience. The focus groups lasted approximately 90 minutes. After the focus group sessions, a demographic questionnaire including questions such as sex and age was filled out by each participant. In the Netherlands, an incentive of €20 was provided to the child and an incentive of €30 was given to the parent.

### Data analysis

Data was analyzed using an inductive, bottom-up approach [37]. A qualitative thematic analysis [37], following the steps of Braun & Clarke (2006) was applied to the interviews using Nvivo12. All focus group interviews were audiotaped and written into transcripts by two independent researchers. In the first phase, before the coding process, the researchers familiarized themselves with the data of the transcripts by repeated reading of the data. Transcripts were not returned to participants for questions or comments. In the second step, the initial codes were generated. To maximize reliability, this was done separately by two researchers (Interviews in Dutch: MD (*child & adolescent psychologist, PhD student, female*) and SF (*Cognitive neuroscientist and obesity expert, PhD*, *female*); interviews in French: AF (*obesity therapist, PhD, female*) and DT (*physiologist, Prof. Dr., male*)). To minimize bias, researchers from different backgrounds analyzed the data. In the second step, the initial codes were generated, and the researchers separately assigned codes to the transcripts. A description of the coding tree was provided. In the third phase, the different codes were sorted by the researchers into potential themes, using visual representations. In the fourth phase, themes were refined and combined via discussion by MD and SF into final themes. Next, in the fifth phase, themes where defined and named and sub-themes were created by the same researchers. In the last step, the findings were written in a report by SF. Data saturation was reached after conducting six adolescent focus groups and four parent focus groups, the same themes occurred. Participants did not provide feedback on the findings.

## Results

Participant characteristics can be found in Table 1 and Table 2.

**Table 1:** Characteristics of the adolescent participants

Adolescents’ characteristics	Belgium (*n* = 5)	the Netherlands (*n* = 10)	France (*n* = 15)	Total (*n* = 30)
Sex				
Male, n (%)	1 (20)	4 (40)	8 (53.3)	13 (43.3)
Female, n (%)	4 (80)	6 (60)	7 (46.7)	17 (56.7)
Age (years), mean (SD)	13 (1.4)	13.6 (1.6)	14.2 (1.8)	13.8 (1.7)
Adjusted BMI, mean (SD)	158.2 (10.6)	136.3 (23.9)	198.3 (46.9)	169 (44.1)

**Table 2:** Characteristics of the parents

Parents’ characteristics	the Netherlands (*n* = 10)	France (*n* = 15)	Total (*n* = 30)
Sex			
Male, n (%)	0 (0)	3 (37.5)	3 (16.7)
Female, n (%)	10 (100)	5 (62.5)	15 (83.3)
Age (years), mean (SD)	45.6 (5.2)	45.3 (5.1)	45.4 (5.2)

Coding of the transcriptions was grouped according to the broad themes and can be found in Table 3. The themes will be reported detailed below for both the adolescents and the parents. The five key themes for both the adolescent groups and parent groups included: 1) healthy behavior; 2) unhealthy behavior; 3) drivers healthy behavior; 4) drivers unhealthy behavior and 5) needs eHealth application. In the parent groups, an extra subtheme was added on 6) difficulties for healthy lifestyle parenting.

**Table 3:** Themes adolescents and parents

Themes adolescents	Subtheme	% Belgium (*n* = 5)	% the Netherlands (*n* = 10)	% France (*n* = 15)	% Total (*n* = 30)
Healthy behavior					
	Nutrition	100	100	100	100
	Physcial activity	100	100	100	100
	Mental health	0	40	53	40
	Sleep	0	50	0	16.67
	Physical health	0	0	67	33.33
Unhealthy behavior					
	Nutrition	80	90	100	93.33
	Physcial activity	60	60	100	80
	Mental health	40	10	53	36.67
	Sleep	0	30	0	10
	Physical health	0	0	60	30
Drivers healthy behavior					
	Internal motivation	40	60	0	26.67
	Environmental influence				
	Parents				
	Nutrition	100	70	0	40
	Physical activity	80	20	0	20
	Siblings				
	Nutrition	20	10	0	6.67
	Physical activity	20	20	0	10
	Friends				
	Nutrition	0	40	0	13.33
	Physical activity	0	40	0	13.33
	The people you love				
	Nutrition	0	0	53	26.67
	Physical activity	0	0	53	26.67
	Social media				
	Motivation	20	40	0	16.67
	Physical activity	0	0	53	16.67
Drivers unhealthy behavior					
	Internal motivation	20	30	0	13.33
	Feelings	0	0	100	50
	Environmental influence				
	Temptation	80	70	0	36.67
	Parents				
	Nutrition	40	80	53	60
	Physical activity	20	0	60	33.33
	Siblings				
	Nutrition	80	30	0	23.33
	Friends				
	Nutrition	20	60	40	43.33
	Social media				
	Nutrition	0	20	53	33.33
	Physical activity	0	10	67	36.67
	Environment				
	Physical activity	0	0	60	30
Needs eHealth application					
	Monitoring				
	Physical activity	40	40	73	56.67
	Weight	0	0	67	33.33
	Information/tips				
	Inspiration	40	60	0	26.67
	Physical activity	0	0	100	50
	Nutrition	0	0	100	50
	Lifestyle	0	0	100	50
	Increase motivation				
	Rewarders	0	20	0	6.67
	Challenges	20	10	0	6.67
	Reminders	0	10	0	3.33
	Setting goals	40	30	80	56.67
	Sharing with friends	40	30	67	50
	Look and feel				
	Personalize	40	20	0	13.33
	Secure chat	0	0	93	46.67
Themes parents					
Healthy behavior					
	Nutrition		100	100	100
	Physcial activity		89	100	94
	Mental health		0	100	47
	Sleep		22	63	41
Unhealthy behavior					
	Nutrition		78	100	88
	Physcial activity		22	100	59
Drivers healthy behavior					
	Internal factors				
	Feelings		0	100	47
	Environmental influence				
	Parents				
	Nutrition: modelling		78	50	65
	Rewarding		33	0	18
Drivers unhealthy behavior	Stress: emotional eating		56	0	29
	Environmental influence				
	Temptation		33	0	18
	Parents				
	Nutrition: modelling		33	0	18
	Friends		44	0	24
Needs eHealth application					
	Monitoring				
	Physical activity		56	0	29
	Weight		56	0	29
	Nutrition		56	0	29
	Information/tips				
	Facts		67	0	35
	Physical activity		0	88	41
	Nutrition		0	75	35
	Lifestyle		0	75	35
	Increase motivation				
	Rewarders		44	88	65
	Challenges		33	75	53
	Setting goals		11	0	6
	Sharing with friends		22	0	12
	Look and feel				
	Personalize		78	0	41
	User friendly		0	63	29
Education healthy lifestyle: difficult			78	0	41

### Adolescents

#### Healthy behavior

When asking adolescents what they believe defines healthy behavior, all adolescents started by talking about the importance of healthy nutrition. The adolescents mentioned multiple times that for healthy behavior it is essential “… to eat healthy” (male, 12 yrs.) and another specific quote given by a male (16 yrs.) was “… especially nutrition, healthy eating for example”. Another topic that was raised by all participants to contribute to healthy behavior was to have enough physical activity: quotes belonging to this are the following: “… to exercise enough” (female, 12 yrs.) and “… having a good physical condition” (female, 13 yrs.). Next to healthy nutrition and physical activity, mental health was also discussed as being an important element of healthy behavior. Mentioned was for example: “… mental health” (female, 12 yrs.) and “It is not only being physically healthy, also a bit mentally” (male, 16 yrs.). A subgroup of the Dutch adolescents talked about the role of good sleep, for example as: “… and still listening to your body I believe, that if you want to sleep that you then also going to sleep and not that you stay busy until 3 AM, no.” (female, 13 yrs.) and a subgroup of the French adolescents discussed physical health (quoted as “not being ill” or “no pain” (male, 12 yrs.) also as being important for adopting a healthy behavior.

#### Unhealthy behavior

When asked what the adolescents believed defines unhealthy behavior, again, nutrition was discussed by almost all participants: quotes as “… unhealthy eating” (female, 13 yrs.). and “… yes, too much overeating” (male, 12 yrs.) or “… products with sugars and fats. Yes, I think that is unhealthy”(male 16 yrs.) were raised. Physical activity was also mentioned by the majority as being important for unhealthy behavior. Here, quotes related to this were: “… do not have any physical activity at all” (male, 16 yrs.) or “… sitting on the couch all day”(female, 12 yrs.). Mental health was raised as being important for unhealthy behavior by a subgroup of adolescents, for instance: “… not listening to your body”. Also, physical health like having “heart problems”, “diabetes” or “smoking” was discussed by many of the French population. Related quotes were: “smoking will generate health problems” (male, 12yrs.) and “diabetes is due to unhealthy behaviors” (female, 13 yrs.).

Sleep by talking about “… sleeping less” was discussed by a few participants in the Dutch focus group as part of unhealthy behavior. Interestingly, the themes defined when discussing unhealthy behavior largely overlap with discussing healthy behavior. Similar themes included: nutrition, physical activity, mental health and sleep.

#### Drivers healthy behavior

We have asked the adolescents what could contribute to maintain healthy behavior. The discussion was raised by asking the adolescents to elaborate about drivers that help in achieving healthy behavior. The drivers of healthy behavior could be divided into the subthemes internal motivation and external/environmental influences. First, internal motivation was discussed as being important for influencing healthy behavior. Internal motivation refers here to all drivers that were discussed concerning their selves. This was discussed by some adolescents, and a quote was for example: “Because I want to lose weight. That’s it. Before, I didn’t want to lose weight” (male, 14 yrs.). Next, external/environmental influences were discussed as influences of the parents, siblings or friends, on both nutrition and on physical activity for the Dutch and Belgium adolescent group. For the French adolescents, parents, family, siblings and friends were altogether classified as the people you love. They were seen as influencers of both nutrition and physical activity. A quote for influences of friends and family on nutrition is “Yes, I think that friends and family play a big role in being healthy and eating healthier. Because, when someone eats healthy, then you also are more like oh, I will take that too.”(female, 13 yrs.) A quote for influences of friends on physical activity is: “... sometimes, as someone wants to play soccer or something and asks if you want to join, then you join”. (male, 12 yrs.) Another classification is the influence of social media as motivator or as a positive influence on physical activity. A quote related to this: “Yes, like I just said to show it with a before and after picture of someone. That could also be motivating. Because I have seen that before on social media a before and after, and then I think by myself like, looks pretty good then.” (male, 16yrs.). Notably was that the external/environmental influences are being overly expressed and being experienced as more impactful, whereas the influence of internal motivation was raised way less.

#### Drivers unhealthy behavior

Hereafter, we have asked the participants: “What makes it difficult to behave in a healthy way?”. With this we discussed the adolescents experiences of drivers of unhealthy behavior. Here, the drivers can likewise be divided into the key-themes internal motivation and external/environmental influences. Here, several subthemes must be added as well. Next to lack of internal motivation expressed like: “Yes I find it difficult to keep my motivation up if it is just, if there is a chance to not do it, then I mostly ending up also just not doing it.” (female, 12 yrs.), negative feelings (e.g., “feeling sad” (female, 14 yrs.), “being stressed” (female, 14 yrs.) was created as an extra category because this was experienced by all French adolescents to have an influence on unhealthy nutrition. Related quotes are: ““I experience uncontrolled eating when being stressed.” (female, 14yrs.), “When I feel sad, I sometimes eat beyond hunger.” (male 11 yrs.) and “Eating sweet and fat food makes me feel better.” (female, 13 yrs. and male 14 yrs.). Regarding external/environmental influences, these can likewise be classified into (negative) influences of the parents, siblings or friends, on primarily nutrition. A quote related to this: “Yes, my parents do have influence sometimes. Because, uhm, they yeah they are not going outside and they are eating quite unhealthy actually.” (male, 12 yrs.). Social media was also discussed among the adolescents to have a negative influence on health by promoting unhealthy nutrition and less physical activity. For example this was mentioned by a adolescent, male, 16yrs.: “I think that social media, actually keep you from doing things, that you sit inside with for example Instagram.”. Another contributor that was discussed as being important as an external driver of unhealthy behavior is temptation of available food, this was mentioned by some adolescents; for example, “Sometimes it just looks too tasty to not take it”(female, 13 yrs.) Notably, more subthemes have been created in the theme drivers of unhealthy behavior compared to drivers for healthy behavior. Next to the similar themes, the influence of negative emotions and temptations were raised as well as drivers for unhealthy behavior.

#### Needs eHealth application

After the conversation about the experiences of the meaning and drivers of (un)healthy behavior, we have discussed the needs for an eHealth application by raising the question: “What does an eHealth application need to promote healthy behavior?”. One of the things discussed as need for an eHealth application several times was the possibility to monitor own behavior, for instance physical activity and weight. Interesting quotes with regard to this were: “Yes, I actually mean a sort of graphic in which you can track how you did it” (male 16 yrs.) and “… That uhm if you have insight in so much percentage to reach your goal or uhm so many weeks or days or something like that, when you have set your personal goal” (male, 16 yrs.). Another theme that was created as needs in an app was information and tips, this was discussed as general inspiration by some of the Dutch and Belgium population and, for the French population this was divided into information on: physical exercises, nutrition and lifestyle. A quote related to this: “… no, but I believe that internet is handy, if you for example eat crisps how much weight you would gain from that if you eat that a lot or just how much calories, or just information about unhealthy and healthy food. What is healthy but also tasty, for example, just tips or something like that.“ (female, 14 yrs.). An important issue raised by many of the adolescents was that the app also should have some motivation-increasing factors like the possibility for setting own goals: “… one important thing missing with such an app is that you sort-of-say cannot give a goal. Because the goal could be different for each person. Three categories you want to be conscious about your health? Or do you want to gain or lose weight. Such things were really missing” (female, 15 yrs.) and sharing with friends like: “… With friends or family or just together. On your own is a bit boring. If you do it with friends it is much nicer actually” (male, 12 yrs.). Also a few adolescents discussed as suggestion for adding to an eHealth application to also send rewards, challenges and reminders via the app. The last discussed topic was the need of a nice look and feel of the app. Also the ability to personalize was discussed by a few, and the possibility of a secure chat were discussed by some adolescents as important needs for a good application. With this secure chat, adolescents meant a chat without access to people outside the app, without possibility to see their post and discussions being shared to others and with the presence of a moderator to control for kind wordings without possible assault.

### Parents

#### Healthy behavior

All parents discussed the importance of healthy nutrition, a related quote was: “… in any case that you have the responsibility that you buy the products. That you take care that you get the healthy food at home. And uhm, that you also prepare it for them if they are still young.”(female, 51 yrs.). In addition, enough physical activity was talked about as one of the most important elements of healthy behavior, a quote is: “… and doing sports, that is what we try to say with going for a walk, uhm just your movement.” (female, 52 yrs.). Mental health was raised as an important factor by French parents only. Quotes were the following: “... to help our kids feel mentally better, above the impact on weight” (Female, 46 yrs.) and “… to show our kids that they are not alone” (male, 48 yrs,.) Another important category for the parents was sleep, for example: “a healthy sleep rhythm” (female, 52 yrs.) and this was raised by some parents. When comparing the focus groups of the parents with the adolescents, interestingly, a large overlap was found with experiences on healthy nutrition and physical activity. Different was that the adolescents also mentioned physical health and the parents did not.

#### Unhealthy behavior

Interestingly, parents discussed fewer contributors of unhealthy behavior during the focus groups than the adolescents. Unhealthy nutrition was mentioned as most important determinant for unhealthy behavior by many, followed by not having enough physical activity. A quote for unhealthy nutrition is: “Next to healthy eating, does she also eats uh mingle-mangle. That is unhealthy, unfortunately.” (female, 40 yrs.). and for physical activity: “…but what the children do, they don’t move.”(female, 39 yrs).

#### Drivers healthy behavior

The drivers of healthy behavior could be divided into the subthemes internal factors and external/environmental influences. As internal factors, some French parents mentioned experiencing positive feelings raised as: “… being more calm and concentrated” (female, 47 yrs.), “… when they are less sedentary and less tired” (female, 46 yrs.).” as an important driver of healthy behavior. In addition, several external/environmental influences were mentioned, including parent (feeding) behavior. The majority of parents referred to the modeling of nutrition as an important factor leading to healthy behavior of the adolescents. A related quote: “… and I think that the focus also need to be on awareness what healthy nutrition is and a healthy lifestyle can do for you.” (female R2, 12 yrs.). A few parents discussed the monitoring of physical activity. In addition, the rewarding effect of healthy behavior was mentioned as well by a few, for example: “Yes, my children are very sensitive for uhm rewards, for something that uhm stimulates to you know. So not always about getting bigger or something but really reward them and yes.”(female, R4 12 yrs.). In terms of parenting style, emotional support was raised as important drivers for healthy behavior by some of the French parents. For example through that parents want to “help to cope with emotions” (female, 46 yrs.) and to “help to improve self-esteem” (female, 47 yrs.). Lastly, positive parenting was discussed by the parents as important motivators for healthy behavior, for example a parent mentioned “… positive feedback and no reproaches” (male, 48 yrs.).

#### Drivers unhealthy behavior

An internal factor that influences unhealthy behavior according to some the parents was emotional eating due to stress. A related quote of a parent is: “… Yes as one of my children is dealing with stress, uhm stress is mostly school related. Uhm then I noticed that they are snitching food more. … (female, 48 yrs.). “External factors were raised by a few parents as the influence of parents and friends on unhealthy nutrition and temptations. A quote related to this: “… And next to this, I also like sweets. So, I have almost every day that I take something, or a piece of chocolate or a croissant with chocolate or something. And yes mostly I don’t act complicated about it, that I tell that I take it myself as well and that I say do you want something as well. “ (female, 52 yrs.). Some of the French parents also experienced that some parenting styles like authoritarian and strict education/parenting could be a contributor to unhealthy behavior: “… restriction and imposition might be counterproductive” (male, 48 yrs.) and “… communication above restriction” (mother, 47yrs.).

#### Needs eHealth application

The first need of an app expressed by some parents is the possibility to monitor own behavior, for instance physical activity, weight and nutrition. Secondly, many parents mentioned useful tips regarding eating habits, physical activity behaviors, and some raised factors related to lifestyle like wellbeing and relaxation as an important feature of an app. A lot of the parents also see opportunities for an app to increase motivation to maintain healthy behavior. The parents mentioned similar elements as their children as for exampling the use of rewards (quote: “by gaining points” (female, 52 yrs.)), challenges, the possibility to set own goals and to share with friends. Quite some parents also expressed the need for an individualized/personalized application. Finally, some parents expressed the need for an easy-to-use application (“user friendly”), which is always available.

#### Difficulty parenting healthy behavior

For the parents we have added an extra theme about the difficulty of parenting healthy behavior. During the focus groups it was noticed that some parents raised experiencing educating a healthy lifestyle as quite difficult. The parents described encouraging healthy behavior as challenging: “For me this is really challenging. Everyday a fight at the dinner table.” (female 40 yrs.) and that it is hard to find a good balance: “I think it is difficult to find that balance and to find a way how you talk about it.”(female, 39 yrs.).

## Discussion

The present study organized focus groups with adolescents with overweight (11–16 years old) and their parents to gain insights into the perceptions of (un)healthy behavior of adolescents with overweight, the drivers of these behaviors, and the needs of eHealth as a tool in treatment. We questioned a heterogenous group of adolescents with overweight and included adolescents from the community, adolescents following an inpatient treatment and adolescents following an outpatient treatment. Although literature had already indicated many variables related to weight gain or the incapacity to lose weight, qualitative interviews, using a bottom up approach, were used in the current study to gain new insights into the problems that are often observed in this clinical group [42].

Our results show that adolescents have well-articulated perceptions on what constitutes healthy and unhealthy behavior and how that may contribute to (un)healthy weight (e.g., acknowledging both nutrition and physical activity), meaning that their narratives correspond to the evidence and findings on what is actually known as healthy or unhealthy habits. A first surprising observation is that these adolescents did not only consider physical health, but also mental health, mentioning factors such as sleep. This finding refers to the need for broadening the perspective: healthy behavior goes much further than just focusing on energy balance. This findings seems to contrast with existing commercial fitness and/or nutrition apps focusing on only one component [34], but it perfectly aligns with a modern view on an integrated biopsychosocial perspective when considering the problem of overweight in young people [43]. In general, the adolescents also have a good perception of the most important internal and external drivers of both healthy and unhealthy behavior. Besides acknowledging the importance of sufficient internal motivation, feelings (e.g. “feeling sad”, “being stressed”) were also recognized as (conflicting) internal drivers. This recognition is in accordance with other studies [23, 44, 45] which reported that people with overweight can experience different triggers (other than hunger) to eat, which should receive sufficient attention. This also means that lifestyle interventions could be improved when they can capture these different triggers. In multidisciplinary interventions, identifying different triggers is already an acknowledged component and often relies on diary reports. However, it requires intensive face-to-face contact to discuss the triggers and learn to cope with them [12]. Moreover, there is often a large time span between the experience of a trigger and the contact with a therapist. Introducing smartphone apps as part of an intervention could offer new opportunities, especially when they include ecological momentary assessments for monitoring and personalization of treatment [23].

Adolescents recognize different external drivers of their behavior, such as their parents, siblings, peers and social media. An interesting observation in this regard is that these adolescents identify their parents as important influencers for both their healthy and unhealthy behaviors, whereas parents mainly mention the positive influence they have on the behavior of their child regarding the adoption of a healthy lifestyle. These results suggest that parents underestimate their role and influence the unhealthy behavior of their child. An observational study obtained similar results in the context of mealtime and its related interactions: parents of children with weight problems report high monitoring and rule setting, while observations of their actual behavior evidence the opposite [46]. The lack of parental awareness questions their role as neutral observers, whereas an app could be a neutral comprehensive monitoring tool. It should be noted that the influence of the parents remains important also in this developmental stage, although the influence of other external drivers (such as social media, peers) definitely increases during adolescence, and is highly rewarding [47]. This finding firstly suggests that new (eHealth) intervention programs for adolescents with overweight definitely also need to involve parents, offer them psychoeducation, and recommend them to be aware of their parenting practices regarding eating behavior and physical activity when a healthy lifestyle is indicated [15, 17, 46]. In this regard, research shows that the parental influence on healthy eating and physical activity is threefold; by specific parent (feeding) practices (e.g., exposure to and availability of food and modeling), by parenting style (e.g., parental warmth and parental support), and by the family functioning [16]. Besides recognizing the importance of parenting through modeling a healthy behavior and adopting parenting practices such as rewarding adolescents’ healthy behavior, this study reveals that parents seem unaware of other parenting practices like limit setting or providing a healthy food environment and of the influence of the parenting style and family functioning on the dietary intake of their child. In addition, results show that parents appear to be unconscious of how they often also model unhealthy behavior or provide too much attractive food. These results emphasize the importance of involving the parents in the treatment of overweight adolescents by sensitizing them about their lasting role during adolescence and their influence on both healthier and less unhealthy behaviors. Consequently, existing commercial fitness and/or nutrition apps as stand-alone interventions will not suffice. However, the parents also mention an important influence of the peers on the unhealthy behavior of their child. Not surprisingly, parents report their parenting as challenging in terms of adopting a healthy lifestyle because of the perceived peer influences. This observation is problematic as it could decrease the parental efforts regarding any help for their child in the indicated weight loss initiatives and even enhance parent-child conflicts. Given that this is not an ideal home environment for adolescents with overweight, it can explain the findings that adolescents often experience parental rejection, which may trigger emotional eating [45, 48]. This finding puts a burden on the parental role as a supportive coach. It seems therefore justified that adolescents with overweight are supported by a tool (or app) that supports them during the day.

It also appears important for adolescents to monitor their behavior and gain insights in the positive and negative influence of different (contextual) factors (peers, siblings, school, social media). So, expectedly, also the adolescents acknowledge that an app would be a strong helpful tool. These adolescents express the need for an individualized approach that recognizes external and internal triggers, provides tips, reminders and feedback, enables to have a (private) chat with someone and to specify individual goals, which they could also share with friends. Developing an app that goes beyond available apps poses some challenges. For example, De Cock et al. [34] reported that the use of available commercial fitness and/or nutrition apps only has a small beneficial influence on adolescents’ snacking and drinking habits. However, thematic analyses of such apps indicated that commercial fitness and nutrition apps tend to incorporate too little behavior change techniques and do not start from research but rather from a one-size-fits-all framework. Given these insights, we specifically need an interactive app that can serve as a comprehensive tool in personalized treatment by mapping individual determinants and mechanisms, which can be responded to in the treatment.

An eHealth app concerning weight loss initiatives is important, but needs awareness of the problem, an underlying level of self-efficacy and health literacy of the user. A limitation of this study is that we recruited motivated adolescents to participate in this study, which limits transferability to all adolescents with overweight. However, the input of 10 different focus groups revealed saturated and very relevant information. The data were provided by different groups of adolescents with overweight from different settings and from three different countries. However, due to the limited sample size, we could not investigate potential differentiation between settings. Another strength of this study is the input of parents, the parents delivered an extra perspective, which gives us unique insights into the problems and needs of their children as well as their own blind spots.

## Conclusion

We can conclude that adolescents have a well-articulated perspective on healthy and unhealthy behavior and their needs, meaning that their narratives correspond to the evidence. In this case, a new app could be of great help. It could function as a day-by-day diary and as a supportive coach. The adolescents expect that the app is interactive and personalized. A personalized tool should take into account the many external as well as internal drivers imbedded in a biopsychosocial perspective on the problem of their overweight.

## Data Availability

The datasets used and/or analyzed during the current study are available from the corresponding author on reasonable request.

## References

[1] Swinburn BA (2019). The global syndemic of obesity, undernutrition, and climate change: the Lancet Commission report. The lancet.

[2] World Health Organization., *Overweight and obesity* 2020.

[3] Rankin J (2016). Psychological consequences of childhood obesity: psychiatric comorbidity and prevention. Adolesc health Med Ther.

[4] Van Vlierberghe L (2009). Psychiatric disorders and symptom severity in referred versus non-referred overweight children and adolescents. Eur Child Adolesc Psychiatry.

[5] Sahoo K (2015). Childhood obesity: causes and consequences. J family Med Prim care.

[6] Organisation WH. *Obesity: preventing and managing the global epidemic* 2000. 11234459

[7] World Health Organization. Global action plan on physical activity 2018–2030: more active people for a healthier world. World Health Organization; 2019.

[8] Chu D-T (2018). An update on physical health and economic consequences of overweight and obesity. Diabetes & Metabolic Syndrome: Clinical Research & Reviews.

[9] Hamilton D, Dee A, Perry I (2018). The lifetime costs of overweight and obesity in childhood and adolescence: a systematic review. Obes Rev.

[10] Steinbeck KS (2018). Treatment of adolescent obesity. Nat Reviews Endocrinol.

[11] Patton GC (2011). Overweight and obesity between adolescence and young adulthood: a 10-year prospective cohort study. J Adolesc Health.

[12] Braet C (2004). Inpatient treatment for children with obesity: weight loss, psychological well-being, and eating behavior. J Pediatr Psychol.

[13] Finegood D, Kahan S, Gielen AC, Fagan PJ (2014). Complexity, systems thinking, and health behavior change.

[14] Leask CF (2019). Framework, principles and recommendations for utilising participatory methodologies in the co-creation and evaluation of public health interventions. Res Involv Engagem.

[15] Campbell KJ (2007). Associations between the home food environment and obesity-promoting eating behaviors in adolescence. Obesity.

[16] Rhee K (2008). Childhood overweight and the relationship between parent behaviors, parenting style, and family functioning. The ANNALS of the American Academy of Political and Social Science.

[17] Tzou IL, Chu N-F. *Parental influence on childhood obesity: A review* 2012.

[18] Al-Khudairy L et al. *Diet, physical activity and behavioural interventions for the treatment of overweight or obese adolescents aged 12 to 17 years*. Cochrane database of systematic reviews, 2017(6). 10.1002/14651858.CD012691 PMC6481371 28639320

[19] Mead E et al. *Diet, physical activity and behavioural interventions for the treatment of overweight or obese children from the age of 6 to 11 years* Cochrane Database of Systematic Reviews, 2017(6). 10.1002/14651858.CD012651 PMC6481885 28639319

[20] Bray G (2004). Obesity is a chronic, relapsing neurochemical disease. Int J Obes.

[21] Jones RA (2011). The importance of long-term follow-up in child and adolescent obesity prevention interventions. Int J Pediatr Obes.

[22] Cardel MI (2020). Obesity treatment among adolescents: a review of current evidence and future directions. JAMA Pediatr.

[23] Spanakis G et al. *Network analysis of ecological momentary assessment data for monitoring and understanding eating behavior*. in *ICSH*. 2015. Springer.

[24] Braet C (2012). Subtyping children and adolescents who are overweight based on eating pathology and psychopathology. Eur Eat Disorders Rev.

[25] Goldschmidt AB (2008). Subtyping children and adolescents with loss of control eating by negative affect and dietary restraint. Behav Res Ther.

[26] Boh B (2016). An ecological momentary intervention for weight loss and healthy eating via smartphone and internet: study protocol for a randomised controlled trial. Trials.

[27] Eysenbach G (2001). What is e-health?. J Med Internet Res.

[28] Lyzwinski LN (2019). Mindful eating mobile health apps: review and appraisal. JMIR mental health.

[29] Bauer K (2020). Conventional weight loss interventions across the different BMI obesity classes: a systematic review and quantitative comparative analysis. Eur Eat Disorders Rev.

[30] Hind J, Sibbald SL. *Smartphone applications for mental health—a rapid review*.Western Undergraduate Research Journal: Health and Natural Sciences, 2014. **5**(1).

[31] Luxton DD (2011). mHealth for mental health: integrating smartphone technology in behavioral healthcare. Prof Psychology: Res Pract.

[32] Shiffman S, Stone AA, Hufford MR (2008). Ecological momentary assessment. Annu Rev Clin Psychol.

[33] Myin-Germeys I, Kuppens P. *The open handbook of experience sampling methodology* The Open Handbook of Experience Sampling Methodology; Independently Publisher: Chicago, IL, USA, 2021: p. 1-311.

[34] De Cock N (2018). Feasibility and impact study of a reward-based mobile application to improve adolescents’ snacking habits. Public Health Nutr.

[35] O’Malley G (2014). Exploring the usability of a mobile app for adolescent obesity management. JMIR mHealth and uHealth.

[36] Browne S (2020). Mobile health apps in pediatric obesity treatment: process outcomes from a feasibility study of a multicomponent intervention. JMIR mHealth and uHealth.

[37] Braun V, Clarke V (2006). Using thematic analysis in psychology. Qualitative Res Psychol.

[38] Tong A, Sainsbury P, Craig J (2007). Consolidated criteria for reporting qualitative research (COREQ): a 32-item checklist for interviews and focus groups. Int J Qual Health Care.

[39] Van Winckel M, Van Mil E (2001). *Wanneer is dik té dik?[When is fat too fat?]* Behandelstrategieën bij kinderen met overgewicht [Treatment strategies in overweight children].

[40] Tyupa S (2011). A theoretical framework for back-translation as a quality assessment tool. New Voices in Translation Studies.

[41] Brislin RW (1970). Back-translation for cross-cultural research. J Cross-Cult Psychol.

[42] Naets T (2020). Adherence and barriers in e-health self‐control training for enhancing childhood multidisciplinary obesity treatment. Clin Psychol Psychother.

[43] Russell CG, Russell A (2019). A biopsychosocial approach to processes and pathways in the development of overweight and obesity in childhood: insights from developmental theory and research. Obes Rev.

[44] Debeuf T (2018). Stress and eating behavior: a daily diary study in youngsters. Front Psychol.

[45] Vandewalle J, Moens E, Braet C (2014). Comprehending emotional eating in obese youngsters: the role of parental rejection and emotion regulation. Int J Obes.

[46] Moens E, Braet C, Soetens B (2007). Observation of family functioning at mealtime: a comparison between families of children with and without overweight. J Pediatr Psychol.

[47] Vandevivere E, Braet C, Bosmans G (2015). Under which conditions do early adolescents need maternal support?. J Early Adolescence.

[48] Vandewalle J (2017). The daily relation between parental rejection and emotional eating in youngsters: a diary study. Front Psychol.

